# ‘You’re not going to give a monkey’s chuff’: exploring co-production in the design of services for women who have experienced sexual violence

**DOI:** 10.1177/17579139221106838

**Published:** 2022-06-29

**Authors:** L Warwick-Booth, R Cross, S Coan, P Fisher

**Affiliations:** Centre for Health Promotion Research, Leeds Beckett University, City Campus, 519 Portland Way, Leeds LS1 3HE, UK; Centre for Health Promotion Research, Leeds Beckett University, Leeds, UK; Centre for Health Promotion Research, Leeds Beckett University, Leeds, UK; Centre for Health Promotion Research, Leeds Beckett University, Leeds, UK

**Keywords:** co-production, evaluation, service design, sexual violence

## Abstract

**Aims:**

Co-production is an emerging field in public health practice. We aim to present evidence of what works well to support co-production and what can be improved based upon learning from our evaluation of a co-production project implemented by Rape Crisis England and Wales (RCEW). RCEW designed and delivered a national co-production project called Weaving the Web, to inform the development of an online support service for women who have experienced sexual violence.

**Methods:**

We qualitatively evaluated the RCEW co-production approach. The specific objectives of our evaluation were to assess the increased role and voice for women and girls in co-producing services and provide better quality of evidence for what works in empowering women and girls. The evaluation was conducted in two phases: Phase 1 was the observation of co-production events (*n* = 8), with findings from this used to develop an interview schedule for Phase 2, where semi-structured interviews (*n* = 26) were conducted with a range of stakeholders (staff, partners and service users).

**Results:**

Staff supporting the co-production project were highly committed to the work, investing time, money, and preparation, and having a good understanding of co-production. Service users were less familiar with the approach and felt alienated by some of the language used. Most service users described participation as empowering and, in some instances, important in their own recovery. They were keen to stay involved beyond the creation of the online resource.

**Conclusion:**

The data from our evaluation illustrate that co-production on a national level is challenging. While RCEW used values-based practice, and provided a supportive culture to underpin the co-production of their online service, transformative engagement and true participation were not achieved. Learning from this project is drawn out here to outline transferrable lessons for practitioners intending to use models of co-production in other public health settings.

## Introduction

The academic literature contains much about the definition of co-production in both service design and research. There is also much discussion about the theory and principles underpinning co-production, but there is less evidence about what it looks like in practice, the realities of delivering it, and the challenges that it can encompass.^1^ This may include expressions of indifference from some participants, as referenced in the quotation included in the title of this article. ‘Monkey’s chuff’ is a slang term from the north of England, used to convey a lack of concern and indifference.

The literature describes benefits for all parties engaging in co-production with ‘intrinsic value’ for individuals from being connected and valued, and an ‘increased capacity and impact’ for services.^2^ The UK government guidance for services relating to violence against women and girls (VAWGs) states ‘good commissioning should begin with an understanding that VAWG survivors are experts in their own lives and are integral to the design of services’. Equally, specialist service providers have a breadth of expert knowledge and experience to draw on. Involving survivors and specialist service providers in the whole commissioning process by way of co-production brings a range of benefits for all of those involved.^3^ As voluntary organisations deliver a range of public services, it is important to evaluate how they incorporate co-production into their work, in particular what works and for whom.

There is often a focus on volunteers as participants in third sector research on co-production, so there is a need to investigate the experience of user involvement in service design. Research suggests that co-production in the third sector is more likely to have an impact on participants when led informally and less bureaucratically. Organisations that present opportunities for service users to be involved and make decisions at a local level, without authorisation from layers of management, have more success.^4^

There are examples in mental health services of co-production leading to more culturally appropriate provision with greater reach to Black, Asian and Minority Ethnic service users^5^ and for individuals who have experienced mental illness, there is evidence that co-production approaches improve wellbeing, reduce stigma and build people’s agency and skills.^2^ For victims of sexual violence, the benefits of involvement can go further still. People who have experienced sexual violence often experience shame and exclusion as a result of harmful societal narratives, but ‘co-production offers survivors the possibility of re-authoring the narratives that hinder change […] and thereby challenging the othering of victim-survivors’.^6^ A trauma-informed approach to co-production in this context views people as assets (not passive recipients) and supports their development and empowerment.^6^ However, in VAWG services in particular, some analysis shows that the co-production employed in developing services is consultation rather than transformatory.^7^ Barriers to co-production in the third sector overlap with those seen in public sector work, particularly around resistance from staff, limited resources and use of jargon, and even if the principles are adopted initially, it is difficult to sustain the practice.^4^

This article considers findings from the evaluation of a voluntary sector organisation’s (Rape Crisis England and Wales (RCEW)) approach using national co-production in the design of services for women and girls who have experienced sexual violence. RCEW wanted the specialised online provision to help to reduce waiting times for support and also to be able to reach women not able to access services in person. In March 2016, the average wait for face-to-face support was 5–6 months, and there were more than 4000 people on the waiting list.^8^

The organisation was committed to working with survivors (this is their choice of language) and staff to develop the platform through co-production events. Co-production was chosen so that stakeholders would have shared ownership of the process of design and delivery of the new online services, with an emphasis on the importance of lived experience informing how services can best meet diverse needs.

The project involved women-only co-production events with staff members and survivors. Specific events were facilitated by specialist partners aimed at reaching underserved groups including Black, Asian and Minority Ethnic women, disabled women, lesbian, gay, bisexual, transgender, queer and intersex (LGBTQI+) women, older women, women from working class backgrounds and young women. There were also online surveys for women and girls, and staff members.

Women and girls’ organisation has a strong track record of service user involvement in the development of support, but as with many attempts at co-production, it usually peaks at intermediate engagement with more buy in needed at a higher level to share power and resources so that it becomes transformative.^7^ This article discusses findings from the evaluation, aiming to provide evidence about what works for co-production to empower women and girls. The authors will examine how the co-production activity was organised and discuss where this fits into the three levels of engagement (descriptive, intermediate and transformative)^9^ and the impact that participation had on the contributors.

## Methods

An external all-female evaluation team was commissioned and funded by RCEW to evaluate their model of co-production from March to June 2017. Our evaluation aimed to evidence the effectiveness of co-production for different groups of women. Our evaluation reported evidence about the increased role and voice for women and girls in co-producing services, as well as reporting upon what works to empower them. Using a qualitative approach, we combined observations and interviews in a phased approach to data generation. Phase 1 was observational. We observed a variety of co-production events in person and online for survivors and stakeholders (internal staff and external partners). During the observations, we used a semi-structured schedule to document the ways in which participants engaged and contributed, as well as the usefulness of the information gathered for the development of online services. We also considered the extent to which the event appeared to provide participants with an ‘enriched environment’ characterised by features such as security, purpose and belonging.

## Summary of Observations

In total, we observed 8 co-production events, with 65 women in attendance. We made notes about each observational setting, guided by our schedule. These notes were thematically analysed, with key themes reviewed and agreed by the research team. On the basis of the analysis of these events, interview schedules were developed in discussion with RCEW staff for use within Phase 2 (semi-structured interviews). Interview schedules were tailored to participant characteristics. Service users were asked about their involvement, contributions to the development of the online platform and their understandings of co-production. Staff and stakeholders were asked about their involvement in the development of online services, their experiences of the project and understandings of co-production. The recruitment of the participants was undertaken by regional Rape Crisis Centres and partner organisations (Tables 1, 2 and 3).

**Table 1:** Regional events held by RCEW

Participants	Date	Location	Number of participants
Service user only event	9 May	Newcastle	3
Mixed (stakeholders and services users)	9 May	Newcastle	10
Mixed	11 May	Cambridge	14
Mixed	20 May	Exeter	17
Service user only event	20 May	Exeter	15

RCEW: Rape Crisis England and Wales.

**Table 2:** Co-production events held by partner organisations

Demographic	Date	Location	Number of participants
Women from working class backgrounds	13 June	London	12
Disabled service users	14 June	Online	2

**Table 3:** External events for women who had not previously accessed rape crisis services

Demographic	Event date	Location	Number of participants
Women who had experienced sexual violence	24 June	Online	2

A total of 26 interviews were conducted, 21 with staff and 5 with service users (Table 4). Interviews were recorded and transcribed for thematic analysis, with findings again agreed within the research team.

**Table 4:** Overview of interviews

Interviews with …	Number of interviews conducted
Interviews with RCEW (Weaving the Web team)	4
Interviews with partner organisations	5
Interviews with centre staff	12
Interviews with service users	5

RCEW: Rape Crisis England and Wales.

Our evaluation received ethical approval through Leeds Beckett University. To ensure ethical rigour, we used informed consent, securing written or verbal consent from all participants. Confidentiality and anonymity were guaranteed in our reporting, and we securely managed information through password protected university systems.

## Results

For the purposes of this article, the data from both phases of the evaluation have been synthesised and brought together under four key themes as follows: (1) understandings of co-production, (2) the value of co-production, (3) what works in co-production and (4) challenges in co-production.

### Theme 1: understandings of co-production

Understandings about co-production varied across the different stakeholder groups. The staff team’s understanding was more apparent. They had experience of working in co-productive ways and were cognisant of the values underpinning these:

*We’ve got a history really of doing bits of co-production. Not necessarily referring to them as co-production but working with service users […] it’s an integral part of the work that [we] do.* (Staff team member)

In contrast, the service users were less familiar with the term, sometimes expressing feeling alienated by some of the terminology that was used. However, one of the service users, who had been involved with the organisation before, described co-production as follows:

*It kind of means […] there are multiple people working on the same team and there are a lot of different ideas and things that are bought together.* (Service user)

The stakeholders’ knowledge of co-production was well developed. In addition, one of them was keen to point out what co-production is not:

*Bad co-production is when you allow someone to believe that it’s co-production and it isn’t. Or […] their views and decisions are only somewhat respected or upheld.* (Stakeholder)

Stakeholder definitions of co-production emphasised the necessity for representation from diverse stakeholders, having shared objectives, trusting the other people involved, working together on an equal footing and removing hierarchy, and giving a voice to those who do not have one. These, in turn, resonate with the values that underpin co-production in practice. During the observations, we also watched how the participants dealt with differences of opinion; while there was implicit agreement at the co-production events that different approaches were needed, there was no significant variance in their delivery, limiting the ways in which spontaneous discussion and disagreement could be used for learning.

### Theme 2: the value of co-production

The process of co-production (being involved) was empowering, and the service users felt proud to participate allowing them to voice their opinions and learn from each other:

*I mean if people can listen to one another then there’s usually something to learn about that other person or sometimes about yourself.* (Service user)

*We bring all our ideas and put it into one basket.* (Service user)

For some service users, partaking in the co-production activities was an additional form of support:

*I felt […] talking is a massive therapy […] so being able to, it felt quite good to be able to tell people exactly what personally I would have wanted.* (Service user)

The staff and stakeholders were also satisfied that the service users had enjoyed the events and were able to positively contribute generating new ideas:

*From the feedback we’ve had … they enjoyed the event. I thought the events had a really good feel to them.* (Staff team member)

*I think they felt that this was a space where they could speak, and they did contribute.* (Stakeholder)

We observed that RCEW created dedicated spaces for alternative and minority perspectives with specific events hosted for women with stated characteristics. Furthermore, the stakeholders felt that their own expertise had also been valued in the project and that the principles of co-production had been enacted in the approach taken – ‘we were contacted for our expertise’ and ‘we have been listened to and very much respected’ (various stakeholders).

### Theme 3: what works in co-production

During the observations, we considered if the participants felt able to express their views openly and honestly. The environment appeared to support openness in the co-production sessions. Body language was generally relaxed and open; in some sessions, service users felt able to disclose their experiences of sexual violence. Interview data confirmed our observations:

*To make the environment as welcoming, as friendly […] just acknowledging that people were doing a favour basically, and that was really valuable.* (Stakeholder)

The content of the co-production events had been generated through lengthy research and discussion with stakeholders, including service users, prior to the events themselves. This demonstrates the process of learning and education that is often required in co-productive working with an emphasis on bringing people together:

*They’ve learnt about the project … and everyone now is at that same point of the journey and I think that’s the huge things about co-production is making everyone equal.* (Staff team member)

Staff had primed the service users in advance to consider themselves ‘experts by experience’ (Staff team member). The service users who took part contributed their ideas to the process demonstrating that they had been well-informed and had been prepared to take part. The importance of power sharing was emphasised:

*I think the thing with co-production … is about sharing the power of it. The power of knowledge of being able to define terms … being able to define what is talked about, what is permissible, what is not …* (Stakeholder)

### Theme 4: challenges in co-production

During the observation of events, we considered the extent to which participants felt able to take a fluid approach, enabling them to revise views. There were no obvious occasions pointing to changes in views. The format of the day was highly structured - more opportunities for spontaneous and unstructured talk might have been helpful.

Our observational data also noted that while participants from all stakeholder groups engaged positively with the process, staff were sometimes more vocal than service users. We observed that attendance at some of the service user events was low, particularly online, despite the fact that RCEW provided high-quality preparatory material. This may be related to geographic obstacles and possibly point to some of the challenges of co-ordinating co-production at local and national levels. For many service users, taking part was not a priority as they have multiple challenges to deal with, so the likelihood is that ‘you’re not gonna give a monkey’s chuff’ (Stakeholder) about contributing to a project such as this.

Challenges also arise when people are unfamiliar with ways of working and ‘weren’t really at the point’ (Staff team member) where they are able to work in fully co-productive ways. One of the challenges for the service users included time. For some, the event felt rushed, and they would have liked more time for reflection:

*It would be been interesting to have been a bit more leisurely, had a bit of discussion … I think we could have gone a bit more deeply … it was too rushed.* (Service user)

Time was an issue also picked up by the stakeholders but in a different way. Concerns were expressed about the amount of time and resources needed for co-production working which is seen as a time-consuming and lengthy process ‘we feel like it has been a long time … when is it going to start?’ (Stakeholder).

Another service user perceived something of a power imbalance in the way that the events were organised:

*It just felt like being in a classroom to be honest.* (Service user)

In addition, the structured format to the event days that we observed limited the extent to which the service users could dictate the course of events and how things could progress. In a few cases, the expertise of the participants may have been overlooked. Finally, service users were keen to be involved more in future but had not been given information about how to be.

## Discussion

This evaluation adds evidence to the existing co-production literature by further illustrating the worth of such approaches for participants, as they can be valuable in terms of the positive outcomes that arise.^2,3^ In this instance, participation was, in many cases, empowering and made the service users feel valued, a potentially important contribution to their recovery from experiences of sexual violence.^6^

In order to be successful, co-production is recognised in the literature as requiring an authentic form of engagement that emanates from a particular set of values.^1^ In this example, the staff team’s long-standing attachment to the principles and practices of co-production was important in engaging service users, and capturing their views. The model of co-production used by RCEW is a good example of the adoption of a bespoke, flexible, partnership approach focusing upon the proactive inclusion of underserved groups. There was also evidence of RCEW creating a supportive enriched environment^10^ through a carefully researched and planned approach to the co-production events. RCEW ensured that women felt safe in the events, essential for those who have experienced sexual assault to be able to contribute. Evidence suggests that supportive cultures in which people feel secure and have a sense of belonging are important underpinnings of successful co-production.^9^ However, despite this supportive culture, fewer service users and women who had experienced sexual violence were involved than RCEW intended. While co-production is assumed to always be good in principle, the reality is that the process and associated expectations do not always marry with what service users want, particularly where experiences of trauma may be revisited.

Co-production in this example was further enabled by an ethos of ongoing learning, and openness to revising positions among the professionals involved. There was, however, tension here between the encouragement of diverse expertise (including different groups of women) and the management of a national co-production project requiring some degree of uniformity to ensure its successful completion.

Further challenges arose in the disconnect between the language of professionals and service users, which can be alienating for participants, and cause discomfort in instances where language is perceived to be inaccessible. While events enabled the mutual sharing of information and learning, they were led using a structured didactic approach, which is at odds with the principles of co-production. Therefore, the full principles of co-production were not met in the RCEW approach. There is evidence of the RCEW co-production model encompassing shared decision-making (descriptive level) as well as acknowledging contributions from clients (intermediate level).^9^ However, the transformatory level of relocating power and control, with significant shaping by service users, is not evident in our evaluation data. Applying a ladder of participation approach^11^ to the RCEW approach, this work was more in line with consultation rather than being located higher up the ladder. Therefore, despite staff commitment to and investment in co-production, RCEW used a model which limited power sharing because professionals ultimately remained in control of both the process and events. There may have been missed opportunities for increased service user ownership of the process and events in line with notions of power sharing.^12^ Those with power should use it to support marginalised populations by providing an environment that enables change.^12^ Finally, there are many models that practitioners can use beyond what is described in this article (events), for example, steering groups or co-leadership designs, which may, in part, address criticisms of tokenism, enhance power sharing and increase participation.

## Conclusion

In summary, there are several transferrable lessons that can be drawn from the RCEW approach to co-production, which are useful for public health practitioners implementing similar work. What worked well for RCEW was their values-based practice, their creation of a supportive environment and their inclusive approach. However, challenges arose as a result of the scale of the work. Co-production on a national level is complicated, and does not always sit neatly with local practice and approaches. Thus, national co-production needs to be recognised as more difficult to implement when compared to local approaches.

While certain guidelines and principles are common to all forms of co-production, it is highly context dependent; therefore, practitioners need to account for the context in which they are working. For co-production to flourish, the environment in which it takes place has to be secure and enabling so that people feel able to contribute. The relational elements of co-production should not be overlooked as all of those involved need to feel valued, have a definition of co-production to work with and be able to use their assets within the process. Parity of expertise is important in addressing power imbalances between professionals and other community members involved, because all knowledge has to be valued.

## References

[1] Warwick-Booth L Bagnall AMB Coan S . Creating participatory research. Principles, practice and reality. Bristol: Policy Press; 2021.

[2] NEF. Co-production in mental health: a literature review. London. Available online at: https://neweconomics.org/uploads/files/ca0975b7cd88125c3e_ywm6bp3l1.pdf (last accessed 11 January 2022).

[3] UK Home Office. Violence against women and girls services supporting local commissioning. London; 2016. Available online at: https://assets.publishing.service.gov.uk/government/uploads/system/uploads/attachment_data/file/576238/VAWG_Commissioning_Toolkit.pdf (last accessed 7 January 2022).

[4] Benjamin LM Brudney JL . What do voluntary sector studies offer research on co-production? In: Benjamin LM Brudney JL (eds) Co-production and co-creation. London: Routledge; 2018, pp. 49–60.

[5] Hatzidimitriadou E Mantovani N Keating F . Evaluation of coproduction processes in a community-based mental health project in Wandsworth. London: Kingston University/St George’s University of London; 2012.

[6] Fisher P Balfour B Moss S . Advocating co-productive engagement with marginalised people: a specific perspective on and by survivors of childhood sexual abuse. Br J Social Work 2018;48(7):2096–113.

[7] McCarry M Larkins C Berry V et al. The potential for co-production in developing violence against women services in Wales. Soc Pol Soc 2017;17(2):193–208.

[8] RCEW. Weaving the web tender: external evaluation partner of co-production – women only. London: Rape Crisis England and Wales; 2017.

[9] Needham C Carr S . SCIE research briefing 31: co-production: an emerging evidence base for adult social care transformation, 2009. Available online at: https://www.scie.org.uk/publications/briefings/briefing31/ (last accessed 28 January 2022).

[10] Nolan M Grant G Keady J et al. New directions for partnerships: relationship-centred care. In: Nolan M Lundh U Grant G et al. (eds) Partnerships in family care: understanding the caregiving career. Maidenhead: Open University Press; 2003, pp. 257–291.

[11] Arnstein S . A ladder of citizen participation. J Am Plan Assoc 1969;35(4):216–24.

[12] Laverack G . Health promotion practice: power and empowerment. London: Sage; 2004.

